# Stakeholder perspectives of piloting pre-hospital COVID-19 lateral flow testing and direct admissions pathway: exploring why well-received ideas have low uptake

**DOI:** 10.29045/14784726.2022.12.7.3.15

**Published:** 2022-12-01

**Authors:** Fiona C Sampson, Fiona Bell, Joanne E Coster, Elisha Miller, Nicholas Easom

**Affiliations:** University of Sheffield ORCID iD: https://orcid.org/0000-0003-2321-0302; Yorkshire Ambulance Service NHS Trust ORCID iD: https://orcid.org/0000-0003-4503-1903; University of Sheffield ORCID iD: https://orcid.org/0000-0002-0599-4222; Yorkshire Ambulance Service NHS Trust; Hull University Teaching Hospital NHS Trust ORCID iD: https://orcid.org/0000-0001-6413-919X

**Keywords:** alternative pathways, COVID-19 lateral flow test, pre-hospital care, process evaluation

## Abstract

**Introduction:**

In January 2021, Yorkshire Ambulance Service and Hull University Teaching Hospitals implemented a pilot COVID-19 lateral flow testing (LFT) and direct admissions pathway to assess the feasibility of using pre-hospital LFTs to bypass the emergency department. Due to lower than anticipated uptake of the pilot among paramedics, we undertook a process evaluation to assess reasons for low uptake and perceived potential benefits and risks associated with the pilot.

**Methods:**

We undertook semi-structured telephone interviews with 12 paramedics and hospital staff. We aimed to interview paramedics who had taken part in the pilot, those who had received the project information but not taken part and ward staff receiving patients from the pilot. We transcribed interviews verbatim and analysed data using thematic analysis.

**Results:**

Participation in the pilot appeared to be positively influenced by high personal capacity for undertaking research (being ‘research-keen’) and negatively influenced by ‘COVID-19 exhaustion’, electronic information overload and lack of time for training. Barriers to use of the pathway related to ‘poor timing’ of the pilot, restrictive patient eligibility and inclusion criteria. The rapid rollout meant that paramedics had limited knowledge or awareness of the pilot, and pilot participants reported poor understanding of the pilot criteria or the rationale for the criteria. Participants who were involved in the pilot were overwhelmingly positive about the intervention, which they perceived as having limited risks and high potential benefits to the health service, patients and themselves, and supported future roll-out.

**Conclusions:**

Ambulance clinician involvement in rapid research pilots may be improved by using multiple recruitment methods (electronic and other), providing protected time for training and increased direct support for paramedics with lower personal capacity for research. Improved communication (including face-to-face approaches) may help understanding of eligibility criteria and increase appropriate recruitment.

## Introduction

In January 2020, the first cases of SARS-CoV-2 (COVID-19) were diagnosed in the United Kingdom (Lillie et al., 2020) and by January 2021 UK hospitals were admitting over 25,000 patients with COVID-19 each day (GOV.UK, 2022). In January 2021, Yorkshire Ambulance Service (YAS) and Hull University Teaching Hospitals set up a pilot to test the effectiveness of the Mologic™ COVID-19 lateral flow testing (LFT) device to identify patients that may be suitable for direct admission to hospital and avoid emergency department (ED) attendance. The pilot was carried out by emergency ambulance paramedics and aimed to support early identification and management of COVID-19 positive patients, to reduce the risk of nosocomial transmission and to support oxygen demand management across the acute Trust sites (Richards et al., 2022). Patients who were identified as LFT-positive for COVID-19 could bypass the ED and be admitted directly to a specialist COVID-19 ward at a different site within the same Trust. Nucleic acid (polymerase chain reaction, PCR) COVID-19 testing was performed on patients at hospital admission regardless of the LFT result. The YAS ambulance crew could also make a referral to the direct access pathway for patients not included in the testing pilot if they already had a community nucleic acid COVID-19-positive result.

Paramedics in Hull and East Riding of Yorkshire were invited to take part in the testing pilot in January 2021 via direct email, staff update email and wider advertising on YAS staff social media. Uptake of testing by paramedics was lower than anticipated, with 18 paramedics responding to the adverts and 12 undertaking the training and participating in the pilot. As of 5 March 2021, 50 LFTs had been performed but recruitment ended due to the decreasing prevalence of COVID-19 and the need for the dedicated ward to re-open as a surgical ward.

NHS England released lateral flow device testing for ED patient pathways in December 2020 and there has been considerable discussion of the strengths and weaknesses of different point-of-care tests for COVID-19 in the emergency setting (NHS England, 2020; Reynard et al., 2022). As part of a service evaluation to understand how the pilot testing was implemented, we undertook a process evaluation involving semi-structured qualitative interviews with paramedic and receiving ward staff to understand reasons for low uptake, perceived potential benefits and risks associated with the testing and understanding of the processes involved in undertaking the testing.

## Methods

### Study design, setting and sampling

We aimed to recruit between four and six participants from each of the following four cohorts: (1) paramedics who did the training to undertake LFT and actively recruited patients; (2) paramedics who did the training to undertake LFT but did not recruit patients; (3) paramedics who were offered but did not undertake the training; and (4) hospital clinicians at the ward receiving the LFT-positive patients.

Research leads at YAS sent an initial invitation email and follow-up email to all paramedics who had received the training (31 March 2021, 10 May 2021), then sent a further reminder via a follow-up presentation of pilot results to Trust staff. The interview study was advertised on the YAS social media page monthly from April to June 2021 and an advertisement was placed in the weekly staff update email. Snowball sampling (Ritchie & Lewis, 2003, p. 94) was attempted by FCS, who asked respondents to ask people who may or may not know about the study. In order to engage with the staff who were less engaged with social media and emails, a research paramedic visited three ambulance stations on two occasions to try to recruit paramedics from groups 2 and 3.

### Data collection

Semi-structured interviews were conducted by one interviewer (FCS) via telephone and recorded using an encrypted digital recorder. Informed consent was gained and documented from participants prior to the interview taking place. Participants received a £20 Love2Shop voucher as a thank-you for taking part in the study.

### Data analysis and reflexivity

Interviews were transcribed verbatim and checked as analysed. Analysis was undertaken by FCS and JEC following the principles of Braun and Clarke (2006) and using an inductive approach to coding and theme development. Transcripts were coded in NVivo (QSR International, Warrington, UK). FCS and JEC are both non-clinical health services researchers. FCS had recently undertaken interviews with paramedics in another project evaluating another pilot intervention in pre-hospital care (Jones et al., 2020) and was therefore mindful of ensuring this did not impact upon analysis. This enabled them to have an independent perspective that was less influenced by preconceptions of response than may be provided by clinical researchers. Due to the timescales of the analysis, we did not consult patient and public involvement representatives during the development of the proposal. Further details about the pilot intervention are detailed in Table 1.

**Table 1.** Pilot recruitment, training and protocol.

Pilot recruitment methods
Paramedics who had previously been involved in ambulance research, who regularly conveyed patients to the receiving hospital site, were invited by email to participate in the testing pilot. Advert placed into the weekly email YAS Staff Update on 15 and 22 January 2021 advising clinicians of the evaluation, with a request to contact the research department to express an interest if they wished to take part. Staff update also available to all YAS staff on the Trust intranet.
Training offered
Virtual online training (taking between 30 and 60 minutes) offered on Microsoft Teams on 13 January 2021 and 14 January 2021. Six staff were unable to attend either training session and were emailed a recorded copy of the training session. Advised to watch the video and email the research team with confirmation that they had watched it alongside any questions or queries. Copy of the study information, appropriate contact details, admission pathway and inclusion/exclusion criteria made available on the Ambulance Service Research app, which participating paramedics were asked to download for quick reference. LFT devices delivered to participating paramedics for personal use for the pilot duration. Paramedics were authorised to commence recruiting patients once they had completed LFT training.
Paramedic
Fourteen paramedic responses to initial email request were received, eight of whom attended MS Teams virtual training session, four accessed recorded version, two did not complete training. Social media advertising resulted in two further enquiries who did not subsequently respond to follow-up or undertake training. The first patient was recruited on 1 February 2021 and the final patient included on 3 March 2021, with this pilot closing to recruitment on 5 March 2021. Twelve paramedics took part in the pilot and recruited a total of 50 patients, with individual paramedics recruiting between 0 and 11 patients each.
Direct-to-ward referral pathway
The direct-to-ward referral pathway was only available Mon–Fri 9 a.m. to 5 p.m., from 20 January 2021. The pathway was available for all patients who met the criteria and tested positive either by pilot testing or prior community nucleic acid testing. Outside of these hours, paramedics could utilise the Mologic swab test but they were subsequently transported to the local ED and not the designated COVID-19 ward if positive.
Inclusion criteria for patient testing
Over 18 years of age. Being conveyed to Hull University Teaching Hospitals, and the paramedic believes that the patient may require in-patient care. Patient competent to agree to the test.
Exclusion criteria for patient testing
Patient unable or unwilling to agree to the test. Patients requiring a time-critical transfer. Suspected base of skull fracture or other facial, nasal or oropharyngeal trauma or injury. Patients who require access to an acute pathway or with the following presentations: trauma or head injury, acute haemorrhage including suspected GI bleeding, new onset unilateral weakness, suspected MI, suspected acute limb ischaemia, acute abdomen and patients likely to die in the next 24 hours. Patients requiring resuscitation-level care, or with oxygen saturations < 92% on air or on supplemental oxygen or with GCS less than 15. Patients who have had a positive SARS-CoV-2 nucleic acid test in the previous 90 days.

ED: emergency department; GCS: Glasgow Coma Scale; GI: gastrointestinal bleeding; LFT: lateral flow testing; MI: myocardial infarction; YAS: Yorkshire Ambulance Service.

## Results

We recruited 12 paramedic participants who agreed to be interviewed: seven from group 1, one from group 3 and four from group 4 (see Table 2). Despite the use of reminders and other methods to widen recruitment, we were unable to recruit further participants within the timescale and did not interview anyone from group 2 – those who had undertaken the training but not recruited patients. We sought to understand reasons for non-participation within this evaluation as well as testing the pilot. Telephone interviews took place between April and June 2021 and lasted an average of 38 minutes (range 17–64).

**Table 2.** Characteristics of participants.

Length of interview	Code	Sex	Group	Role*
32 mins	LFT1	M	1	Clinical supervisor
38 mins	LFT2	M	1	Newly qualified paramedic
38 mins	LFT3	F	1	Newly qualified paramedic
60 mins	LFT4	M	4	Senior doctor
46 mins	LFT5	F	1	Newly qualified paramedic
34 mins	LFT6	M	4	Junior doctor
51 mins	LFT7	M	1	Paramedic
64 mins	LFT8	F	1	Paramedic
17 mins	LFT9	F	4	Nurse
21 mins	LFT10	F	1	Newly qualified paramedic
21 mins	LFT11	F	3	Paramedic
27 mins	LFT12	F	4	Senior doctor

*Newly qualified paramedic defined as < 2 years in role. LFT: lateral flow testing.

**Figure 1:**
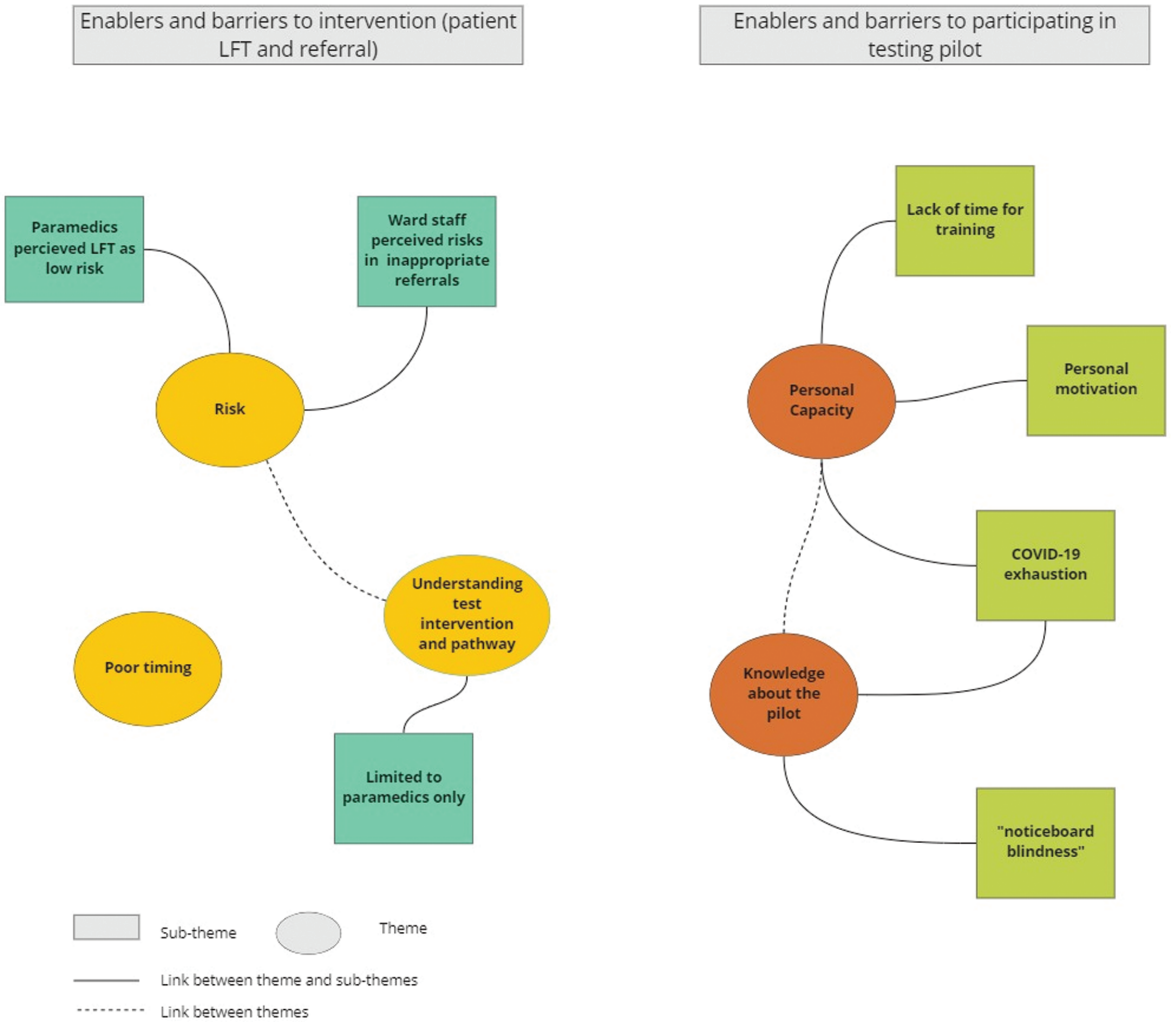
Figure 1. Thematic map of the findings.

Within our findings, we distinguish between barriers and enablers to the intervention (LFT), and barriers and enablers to participating in the pilot. This information was used to determine how the intervention may be adapted or used in future, and where such limitations were as a result of the research process. Figure 1 presents a thematic map of the findings that are presented in this section.

LFT: lateral flow testing.

### Enablers and barriers to the intervention (testing and referral)

#### Participants were positive about the potential for both the lateral flow testing alone and the combined lateral flow testing and pathway

Participants were overwhelmingly positive about the intervention, which they perceived as potentially highly beneficial to the health service, to the patient and to themselves, with limited associated risks. Although not all had been able to use the pathway, they described a wide range of potential benefits of the pathway, including reducing demand on overwhelmed EDs, reducing ambulance waits outside ED and faster, more appropriate care for the patient with potential reduction in nosocomial spread. Only one interviewee referenced the need to reduce the oxygen burden on the main hospital site, which was cited as a principal reason for setting the service up initially.

I think the patient themselves would benefit because they get more specialist care sooner. And then I think the Trust itself benefits because you don’t have all the steps of getting the patient where they need to be. You free up A&E for those patients who really need to be, who are really unwell. You free up the general medical teams that don’t need to look after a standard COVID patient and you help us because we see them first and we do things the way we want to do them and don’t have to try and play catch up. (LFT6)

Yeh if it’s gonna keep the pressures off the emergency department then yeh I think it should be. We don’t know until we try do we, so we’ve got to keep trying and see if it works. If it does take some of the pressure off then I’m all for it. (LFT2)

Paramedics also described additional benefits that they had observed in undertaking the LFT itself (i.e. separate to the pathway) and undertook LFTs even outside the opening hours of the pathway. They perceived the LFT as a quick, simple test that could offer patients appropriate care if results were positive and the speed of results enabled them to relay results back to families to enable immediate isolation. Negative results appeared to offer significant reassurance both to patients who were anxious about COVID-19 and to paramedics who described the anxiety of working around COVID-19.

I think we benefited as well knowing that we’d actually been in contact with a COVID patient. It made me a lot more aware of going home and getting out of my uniform before contact from my family. (LFT8)

I think sometimes it’s piece of mind as well for patients as well, cos obviously we go to a lot of people with a lot of health anxiety about the situation going on and if they were symptomatic and if the lateral flows were coming back negative, which I know is not 100% accurate or anything like that, but it does sometimes give a little bit of reassurance for them as well, which often helps. (LFT11)

Although some participants referenced the LFTs as not being gold standard tests, they provided examples of how the LFT results changed their behaviour and management of the patient, not solely related to use of the pathway. Notably, they described how they would take additional precautions in response to a positive result (e.g. improving airflow in the ambulance, wait outside the ambulance upon arrival at ED, taking additional precautions when returning home from shift).

I think we benefited as well knowing that we’d actually been in contact with a COVID patient. It made me a lot more aware of going home and getting out of my uniform before contact from my family. (LFT8)

We, what we tended to do was if, if we had somebody, like the one that I took in who was positive, we ended up having to wait two hours with him in the back of the ambulance [. . .] so what we’d do is, we’d open the doors of the ambulance, the back doors, and we’d stand outside so therefore you’ve got a flow of air going through the ambulance and dispel anything, and we used to leave the extractor fan going so when we were on the way in with the patient, in the back even though we had the PPE on we had the extractor fan going. (LFT7)

#### Participants perceived the lateral flow testing intervention as low risk

Participants expressed few concerns about risks associated with the intervention and both paramedics and ward staff perceived the intervention as low risk. Some paramedics expressed concerns about the general public hearing that the tests were available and ‘abusing the system’ by calling 999 inappropriately (as LFTs were not publicly available at the time), although some felt it offered an opportunity to help to contain the spread of the pandemic, by helping people who would otherwise struggle to get a test. For paramedics, there was some potential risk in undertaking the test itself, particularly in a moving vehicle. Although they described the test as simple to carry out even on the move, two of the participants identified the safety risk of undertaking the test while the vehicle was moving.

If it gets a bit on social media then obviously, you know people just start ringing up because they want one because they can’t be bothered to go out and get one (yes). That’s the only negative side to it I could possibly see. (LFT7)

Obviously there’s the risks of sort of some sort of soft-tissue injury with having performed a lateral flow test but they’re obviously minimal, we’re doing them twice a week, you know, and most of the country is nowadays aren’t they with most workforces and schools and everything like that. So, I think the risks are quite minimal to be honest. (LFT11)

I think asking for it to be done in a moving ambulance may have risk to the clinicians that are doing it, just because of getting up during transport and trying to sort out the bits of kit. So what I did was just before we set off was do this test and then wait for ten minutes while travelling. So that I’m limiting my exposure to falling in the moving vehicle. (LFT2)

For ward staff, the risks related to the COVID-19 receiving ward not being co-located with specialist services, and concerns about patients being referred to the pathway inappropriately or deteriorating after referral. Although the pathway had been set up to mitigate risks by using strict exclusion criteria to prevent patients with co-morbidities from attending, ward staff still had concerns about patients being inappropriately referred. Only one participant expressed concerns about potentially inaccurate test results, and the impact of admitting someone without COVID-19 to a COVID-19 ward erroneously.

So, there is all those risks that you don’t do the assessment and telephone assessment properly, and there is always risk of getting a patient who’s actually more unwell than it sounds. Now, obviously, there is also the risk of getting a patient crashing and collapsing on the way to the ward, and an infectious diseases ward, it is a bit, in theory, less equipped to deal with a very severe case compared to A&E. [. . .] I can’t think of any other disadvantages to be honest. (LFT12)

I think some of the risks were not getting a full picture over the phone. So I think there was one or two where the ambulance service either saw them and you didn’t get quite a full picture and then they turn up and they’re much sicker than they are. Or in the intervening time where they were quite well with the ambulance initially, by the time you actually see them, when they’re actually on the ward, they’ve become sicker. (LFT6)

I think it was just a tough call for them, you know do they bring them here and then risk them deteriorating and have to have them across town and the implications that that could have, or do they take them direct to A&E where everything’s there should they deteriorate. (LFT9)

#### ‘Poor timing’ resulted in limited opportunity for use of lateral flow testing or the pathway

Barriers to use of the pathway related principally to ‘poor timing’ of the intervention, limited numbers of eligible patients due to falling prevalence (end of UK second wave), limited availability of the direct-to-ward referral pathway (9 a.m.–5 p.m., Monday–Friday) and early closure of the pilot. The short timescale of the pilot was perceived as a barrier for both paramedics who felt that the window of opportunity to sign up to the trial was too short, and for hospital employees who felt that the pathway had not had sufficient time to bed in and make a difference before it had to be closed. Participants felt the intervention would have been of more benefit if started earlier.

I think it’s just timing and I think it took a while for kind of the message to get out to the paramedics that were here. But I think once it got out there were a good number of calls. But I think there still weren’t, I don’t think it was still fully adopted but then by the time it was, that’s when the pandemic was easing and we were closing the wards that we were like taking direct admissions to. And then we lost the place to take direct admissions to. (LFT6)

There was an email sent out about the uptake of patients. And my response was that I was generally not seeing those patients anymore. So that was obviously a big factor. Coming back to your sort of barrier question, maybe there wasn’t enough sort of paramedics doing the trial. Maybe that was a reason why uptake wasn’t that good. Yeh so I definitely felt that maybe two, three weeks since the trial had begun I was seeing less and less of those patients. (LFT2)

#### Understanding the test intervention and pathway

We identified that participants did not have a cohesive understanding of the pilot criteria for use of both the LFT and direct admissions pathway, or the reasons behind the criteria. Even within the small sample that we interviewed, there was evidence of different criteria being used to undertake the LFT, and how the direct admissions pathway operated. Specifically, there appeared to be some confusion about whether the LFT should be undertaken for patients who were symptomatic or for any patients who may require hospital admission regardless of whether they were symptomatic.

Well, we got told that we would do it for anyone going into hospital, it didn’t have to be symptomatic, so, so that’s what I did. (LFT10)

So I understood it that we were testing people prior to going into hospital if we thought they had COVID symptoms. (LFT5)

Similarly, participants described restrictive criteria for referral to the ward, and had limited understanding of reasons behind the exclusion criteria. In particular, many paramedics were unaware of exclusion criteria related to the lack of specialist facilities co-located with the COVID-19 ward site, or the need for consent to undertake LFT within the pilot, which precluded patients’ reduced capacity, for example patients with dementia.

If they had COPD, breathing difficulty, things like that, you can’t swab them and we did go to quite a lot of people with COVID with low sats so it ruled them out completely then! So but if I maybe had more swabs maybe I would have swabbed them but it wasn’t in the criteria so where I would stand on there is different. Because of the study it might have just swayed the results maybe a little bit. (Interviewer: In what way?) Well because, because they aren’t in the criteria, so I don’t know if the study was just purely for people with sats above 92%. If I’m swabbing people below 92% its, it might sway the study a little bit more. (LFT5)

You know I wouldn’t do it on somebody who’s like having a stroke because it’s time, you know they’re time critical and things so it’s, you know, as much as you’d probably want to do one, to rule everything out [. . .] If you’ve got time-critical patients, you don’t do it. If it’s going to slow it down, you know if it’s going to slow you down you don’t do it. You just do it if you feel the time’s there, the patient’s stable and what you think, you know the criteria that you’re happy to fit in. (LFT7)

### Enablers and barriers to participating in the testing pilot

Low uptake of the pilot was considered to be more due to lack of understanding about the trial than because people were opposed to the principles of the trial itself. Participation in the pilot was influenced by personal capacity for undertaking research, ‘COVID-19 exhaustion’ and limited knowledge about the pilot.

#### Personal capacity for undertaking research or innovation

We characterised the paramedic participants within this sample as having a high personal capacity for undertaking research or innovation. They were disproportionately recently qualified and interested in taking part in research or evaluation to understand how healthcare could be improved. Paramedics within this sample were very positive about using the LFT and direct admissions pathway, and perceived the use of diagnostic testing and identification of alternative pathways as an integral part of their role. They were keen to continue the expanding scope of practice for paramedics and saw that expanding pathways could help provide wider benefit for the health service as well as the patient.

Well, you know, it was pretty much the height of sort of COVID-19 and if there was something that I could be doing a little bit extra to sort of keep the public safe or sort of better treatment options then I was willing to sort of undertake that responsibility. (LFT2)

I’m obviously fresh out of university so I’m still quite interested in the research side of everything. And I’m, I’m more than happy when it’s some, comes to a national pandemic where people aren’t quite sure what’s more reliable than anything else, more than happy to do anything to help. (LFT3)

But, anything like that has always been talked about and, and even at university we’re always talking it because I think it’s the, the job roles heading in that direction now where it, it’s not just somebody who takes someone to hospital anymore it’s a decision making and, and more tools that can help with that decision making it is a really positive thing and, and will help the job role grow as well. (LFT10)

Personal capacity to undertake research or innovation appeared to have been negatively impacted by the increased workload and pressure put on emergency services during the pandemic. We conceptualised this as ‘COVID-19 exhaustion’ where the pandemic had generated a higher than usual level of anxiety and burnout that may have prevented paramedics from undertaking anything perceived as ‘extra’ to their everyday work. Although participants reported that the amount of work required to participate in the testing pilot was not significant, some considered that the paperwork involved may have been a factor in preventing people taking part, particularly when the benefits of undertaking the testing pilot were not immediately clear. While participants within this study accepted the work required as a trade-off for the potential benefits to the wider health service, they recognised that this may not be the case for everybody.

I think there were a couple of people that signed up from it but I think if you’re going to ask me the question of why people didn’t sign up for it, for the testing, I think the reason was it was a very, it hit a very anxious time for all of us. Especially in our area I think the lateral, that testing came in just as we’d lost two paramedics to COVID. So I think there was a lot of high anxiety among the staff at the time. And there was people going off [sick] left, right and centre. (LFT8)

I’m not quite sure really, like I said I think people at the time felt as though they had enough to do without having another intervention at the time. Because we were very tired, we were working very, very hard at the time. (LFT5)

Although we were unable to recruit paramedics from the group who did not participate in the pilot, participants referred to ‘old school’, usually a more experienced category of paramedics who may have been less likely to take part in research studies. This was due to them feeling overwhelmed by the existing volume of work and not wanting to take on anything ‘extra’ such as a research study, or potentially even undertake the LFTs themselves.

I think we’ve got quite a lot of sort of older school paramedics and things like that who just, we’ve got a lot of disillusioned staff at the moment. (Interviewer: Yeah, is that since COVID or just generally?) Yeah and then we’ve had management restructures and things like that sort of everyone’s sort of lost a bit of morale at the moment. (LFT11)

I don’t know if it’s because I’ve sort of, we’ve sort of been through that university and like finding out about research but I do find that a lot of sort of older school paramedics don’t look at research in the sort of same way. And don’t get me wrong, some definitely do and I hear a lot from them about it, but I think sometimes it’s more of a case of a lot of people just end up taking patients to hospital cos it’s a lot easier. (LFT10)

A significant barrier that was mentioned by the majority of paramedics was the lack of time available for training and continuing professional development, with paramedics no longer having ‘stand-down time’ in the ambulance station to catch up on emails or training. Although the training was described as short, clear and accessible, none of the participants who mentioned the training had been able to access it within their work shifts. Some paramedic participants within this study appeared to accept the inevitability of undertaking this extra work within their own time, but it was also felt to be a barrier to those with limited personal capacity to undertake the research.

So we did, we did do the, the, the training and everything in our own time which is probably another thing people don’t like to do. Maybe that was another thing I don’t know. So if it helps, if it helps people, patients, an hour or so out of my own days is not a problem at all. (LFT5)

I think one of the things [that might improve recruitment] would be training in, in our, in like job time instead of doing it in your own time, a lot of people wouldn’t do it in their own time I don’t think. (LFT10)

#### Knowledge about the pilot

Paramedics felt that limited awareness of the pilot was a significant barrier to participation. Due to the rapid implementation of the pilot, the sign-up period was brief and communications about the pilot were undertaken mainly via electronic sources (e.g. email, Facebook) and easily missed. Participants reported that other colleagues showed an interest in the study and appeared to be positive about it, but missed the invitation letter and communications about the pilot.

And there were a couple of times when I was doing the, the lateral flow testing and someone’s going, ‘Ooo what’s that?’ and I’m going ‘We’re doing this pre-hospital at the moment just as a trial’. And they were going, ‘I didn’t know anything about that’. So it’s, it’s difficult isn’t it cos you don’t know we, we get emails all the time we, we’re bombarded with emails, at some point every day we get Coronavirus emails, then we get another email and then we get another email so . . . (LFT5)

The impact of the short sign-up period was confounded by the overwhelming volume of information that paramedics receive meaning recruitment emails could easily get lost, particularly given that they were often catching up on emails in their own time and had no time to read them. Paramedics described ‘noticeboard blindness’ and email overload which had worsened during COVID-19 due to an increase in communications and updates. The reliance on e-learning and electronic communication was also a potential barrier for paramedics who were less comfortable with technology, who may be the ‘old school’ group referenced above.

And I think it was more, more people could see it cos like I say a lot of people don’t check their emails as regularly. I do again because, obviously, cos I’m new and it hasn’t broken me yet. (LFT3)

I think also kind of just getting it out to crews. We have to be careful about information overload because we get, you know crews get battered on a daily basis with COVID updates and they do get, there’s a certain amount of noticeboard blindness which comes into play. (LFT1)

### Future implementation and enablers

Participants were overwhelmingly in favour of expanding the use of both lateral flow tests and the pathway in future and felt that other colleagues who had not participated in the pilot would be happy to use the lateral flow tests as another ‘tool in their armoury’. There was support for the use of LFT as standard practice, outside of the trial criteria, and particularly a desire to see the inclusion criteria expanded, both in terms of who could receive the LFT but also expansion of the pathway hours and criteria.

I just thought, I thought if it comes back as a good thing to do then it could, you know it could hopefully be rolled out on a permanent basis things like that, as a standard observation if, if you needed, thought you needed to do one. (LFT7)

We, [sighs] it’s, it is difficult, because the trust pathways are complicated. But, I just, I just think it’s, it’s the way to go. I think why wouldn’t you make a diagnosis as early as possible because then that simplifies the patient’s journey, and that simplifies the work of the hospital. (LFT4)

Paramedics expressed a desire to expand the criteria for inclusion, seeing potential benefits to undertaking testing in the community in order to reassure patients or while they were positive enable them to isolate and follow the guidelines. Three of the paramedics reported that technicians in their crews had expressed an interest in taking part but were unable to and questioned why the LFT could not be done by technicians or a lower acuity team, which would expand the number of crews who could offer testing in the community.

It’s very easy, it was a very easy like pathway to use and I think, I don’t even think just being a paramedic or whatever, it could just as an ECA or a technician role would be able to, would be able to do something like that without any qualms, you know, there’s a lot of like ECAs that are more than capable of doing things like this, you know it wouldn’t necessarily, I wouldn’t see it just as a paramedic role. (LFT7)

And working as a team, you know, it made the whole thing a lot quicker. You know so it was it was, but also she, we’d both done training together to do the swabbing and I didn’t, I wasn’t really sure why it only went out to paramedics. Why couldn’t an ECA do it? (LFT8)

Participants felt that there was scope for improved implementation of similar interventions in future, using multi-faceted recruitment methods and improved communication to increase recruitment. Participants recognised recruitment as a sustained process that needed a longer time period than the pilot had allowed. Importantly, cascading information via nominated research leads, pathways champions or just through seeing other people being involved was felt to be key to improving knowledge of the pilot and thereby increasing recruitment. Multiple methods of recruitment, including non-electronic methods (e.g. posters on noticeboards, cascading via team leaders) and more face-to-face discussions and interactions with the more ‘old school’ group of paramedics, may help prevent problems encountered in recruitment for the pilot.

I think if we’d sort of hammered it to death that we were doing this research that a lot of people would have got involved and I think if we’d have advertised it on stations where people don’t have to check their emails, again that’d probably have got a lot more people involved with it. And obviously there’s a sort of, once somebody knows about it on a station so a lot of people tend, a lot of stations have leads in certain areas. (LFT3)

I still had two crews backing me up, as if it was so I was able to demonstrate to the two crews and cascade that information down like about the possibility of doing lateral flow testing on scene but you know, they all agreed like, it just made sense. (LFT1)

Improved communication about the purpose of the study, what the training and intervention involves and particularly improved understanding of the potential benefits may also help to persuade potential participants that the benefits will outweigh the risks and effort involved.

Another thing I would think would be to sort of explain why we’re doing it so like some people might not, they might think, you know, why have we got to do this, you know, if it was sort of relayed back that, you know, this would change where people might go, you might change the treatment, you know, then it might be more helpful for people to understand why it’s actually been done. (LFT10)

I think a lot of people don’t understand that being involved in a research project isn’t necessarily a massively time-consuming thing it, it doesn’t have to be, you know, the training isn’t necessarily a long thing and it’s just doing what’s best for the patients sometimes. But, a lot of people think it’s a lot more work than it should be. (LFT11)

## Discussion

Our research indicated that pilot participants perceived the intervention as having limited risks and high potential benefits to the health service, patients and themselves. They were overwhelmingly positive about pre-hospital LFT and the alternative pathway in principle, although there had been limited use of the pathway itself due to the timing of the pilot, restrictive patient eligibility and inclusion criteria. Participants supported future expansion of both LFTs and the pathway. They felt frustrated, however, that the intervention had not been implemented earlier and considered that colleagues who had not participated in the pilot would be happy to use the LFTs as another ‘tool in their armoury’ at a time of extreme system pressure.

Despite positive perceptions of the intervention, interviewees identified barriers that resulted in low uptake of the pilot, mainly related to limited knowledge and awareness of the intervention. Participation in the pilot was positively influenced by personal capacity for undertaking research (being ‘research-keen’) and negatively influenced by ‘COVID-19 exhaustion’, electronic information overload and lack of protected time for training. Ironically, the pandemic itself may have reduced capacity for paramedics to take part in the pilot due to exhaustion and a reluctance to be involved in anything extra relating to COVID-19.

The speed of rollout meant coherence and understanding of the intervention was low. Inadequate understanding of an intervention may affect normalisation and take-up of an intervention (Murray et al., 2010). However, within this sample, differential understanding did not appear to influence ‘keenness’ to participate and undertake the LFTs. Uptake may have been higher if paramedics who were less research-keen perceived the benefits of the study to outweigh the difficulties in taking part, particularly at a time where personal capacity for undertaking additional activities was reduced. Improved understanding of the simplicity of the intervention and requirements of the study may have helped to persuade further paramedics to enrol. Wenke et al. (2020) similarly identified that beliefs about positive consequences or research and emotional responses of being overwhelmed influenced participation in research in a study of allied health professionals.

While LFT pilots have taken place in other ambulance services (South Central Ambulance Service NHS Foundation Trust, 2021), we are not aware of any other studies reporting on the feasibility of using LFT to support alternative pathways. Evidence suggests that paramedics, patients and healthcare practitioners support alternatives to direct ED conveyance, although existing evidence relates primarily to non-conveyance rather than conveyance to an alternative location (Blodgett et al., 2021). Other studies have identified factors affecting participation in pre-hospital clinical trials and embedding a culture of research, highlighting the importance of peer communication in recruitment, rather than electronic communication (Pocock et al., 2019). Studies evaluating experiences of and challenges to taking part in pre-hospital research have focused principally on the experiences of paramedics who have recruited patients to clinical trials and the ethical challenges faced (Ankolekar et al., 2014; Armstrong et al., 2021; Lazarus et al., 2019; Pocock et al., 2019). While these studies have similarly identified the high level of motivation of research-active paramedics, there is a clear need for further research exploring barriers to participation among paramedics who are not research active and particularly to understand what might motivate paramedics who are not currently research active to become so.

## Limitations

This was a process evaluation of an intervention in a single setting, which carries limitations to the transferability of the findings to other settings. Many of the problems associated with the pathway related to the location of the COVID-19 ward which significantly limited the inclusion criteria and accessibility of the pathway, which would be different for a ward that was co-located with the ED/ICU. Although we spoke to Hull University Teaching Hospitals staff about the pathway, and paramedics about the LFT, we did not get an adequate sample who could talk about the actual handover and delivery of patients to the pathway. Similarly, despite several attempts, we did not manage to recruit any paramedics who did not sign up to the pilot, which limits the interpretation of results. The overall sample size was low, although we did achieve a sample size of 12 which is considered by some as the ‘magic number’ at which saturation of themes starts to occur (Guest et al., 2006), and a systematic review recently identified that saturation can occur at between 9 and 17 interviews (Hennink & Kaiser, 2022), although the concept of saturation in qualitative research is contended (Braun & Clarke, 2021). While it was not possible to determine if saturation had occurred from 12 interviews, similar findings were identified from the later interviews, suggesting that saturation was occurring at some level.

The analysis was necessarily descriptive, partly due to the small sample size, but also due to the purpose of the study (to provide lessons to the ambulance service in undertaking similar interventions in future). Although elevating qualitative analysis to the interpretative level can provide more in-depth insights, there is value to undertaking more simple, descriptive analysis (Broom, 2021).

## Implications and conclusions

This process evaluation suggested that paramedic use of LFTs to identify patients suitable for alternative pathways may be welcomed by both pre-hospital and ward staff, although expanded criteria and receiving ward availability may enable the intervention to be more widely adopted. Recruitment to pre-hospital rapid research pilots may be improved by using multiple recruitment methods (electronic and other) and providing protected time for training and support for paramedics with lower capacity for research. In particular, identifying ways of highlighting research pilots that do not rely on electronic methods may be key to overcoming electronic information overload. Improved communication including face-to-face approaches and ambulance station research champions may help understanding of eligibility criteria and increase appropriate recruitment. Expanding the eligibility criteria to enable technicians to participate as well as paramedics may help with roll-out and coverage of the intervention as well as expanding awareness.

## Acknowledgements

We would like to thank all research participants for giving up their time to speak to us, Kelly Hird and Michael Hooker at YAS for help with recruitment and Marc Chattle for help with arranging transcription of interviews and payment of participants.

## Author contributions

FCS contributed to study design, undertook data collection and analysis, drafted the article and approved the final version. FB and NE conceived the study, helped with the study design, contributed to analysis of the data, critically revised the article and approved the final version. JEC and EM contributed to acquisition, analysis and interpretation of the data, critically revised the article and approved it for the final version. FCS acts as the guarantor for this article.

## Conflict of interest

Lateral flow test devices were donated by Mologic UK Ltd. No funding was received from Mologic UK for this or any other work. Mologic UK did not contribute to study design, analysis or reporting of the results.

## Ethics

Ethical approval was granted from the University of Sheffield ScHARR ethics committee (UoS 038599) on 31 March 2021. The study was classified as a service evaluation and therefore research governance approval was not required.

## Funding

This project was supported by the National Institute for Health Research (NIHR) Clinical Research Network Yorkshire and Humber using UK aid from the UK Government to support global health research. The views expressed in this publication are those of the authors and not necessarily those of the NIHR or the UK Department of Health and Social Care.
